# Epidemiological Shifts in Respiratory Virus Infections Among Older Adults (≥65 Years) Before and After the COVID-19 Pandemic: An 18-Year Retrospective Study in the Republic of Korea

**DOI:** 10.3390/microorganisms13102301

**Published:** 2025-10-03

**Authors:** Jeong Su Han, Sung Hun Jang, Jae-Sik Jeon, Kyung Bae Lee, Jae Kyung Kim

**Affiliations:** 1 Department of Biomedical Laboratory Science, College of Health Sciences, Dankook University, Cheonan-si 31116, Republic of Korea; jshan1162@naver.com (J.S.H.); zenty87@naver.com (J.-S.J.); 2 Department of Medical Laser, Graduate School of Medicine, Dankook University, Cheonan-si 31116, Republic of Korea; well8143@naver.com; 3 Research Center for Bio-Functional and Biocompatible Materials, Dankook University, Cheonan-si 31116, Republic of Korea; 4 Department of Medical Laboratory Science, Seoyeong University, Paju-si 10843, Republic of Korea; gurake@hanmail.net

**Keywords:** COVID-19, epidemiology, older adults, respiratory viruses, seasonality, SDG 3, sex differences, surveillance, universal health coverage

## Abstract

We investigated respiratory virus epidemiology in older adults across pre-pandemic (2007–2019), pandemic (2020–2022), and post-pandemic (2023–2024) periods, focusing on how public health interventions shaped surveillance, prevalence, and sex-specific trends. We conducted a retrospective cross-sectional study at a 1000-bed tertiary hospital in the Republic of Korea during 2007–2024, analyzing 4692 nasopharyngeal swab specimens collected from adults aged ≥ 65 years with suspected respiratory infections during 2007–2024. The specimens were tested for 15 respiratory viruses using multiplex real-time polymerase chain reaction. The outcomes included virus-specific detection rates and seasonal, sex-based and temporal trends before and after the COVID-19 pandemic. During the pre-pandemic period, older adults accounted for 13.2% of the tested individuals, which significantly increased to 52.0% in the later periods. *Influenza A* was the most frequently detected virus, followed by rhinovirus and human metapneumovirus. *Influenza, RSV A/B*, and *coronaviruses 229E* and *OC43* showed peak positivity in winter, *parainfluenza virus type 3* peaked in summer, and *rhinovirus* circulated year-round. Virus circulation was markedly suppressed during 2020–2022 and partially rebounded during 2023–2024. This study highlights the shift in diagnostic access and epidemiologic patterns of respiratory virus infections in older adults following the COVID-19 pandemic.

## 1. Introduction

Older adults (≥65 years) are particularly vulnerable to respiratory viral infections due to immunosenescence and the high prevalence of chronic comorbidities in this population [1,2,3]. These infections are associated with increased risks of severe illness, hospitalization, and mortality, highlighting the importance of early surveillance and prevention efforts [4]. Consequently, the Centers for Disease Control and Prevention has prioritized influenza vaccination and surveillance in older adults [5].

Respiratory viruses encompass several pathogens that are responsible for clinical manifestations ranging from mild upper respiratory tract infections to severe pneumonia [6]. Influenza viruses, negative-sense, single-stranded RNA viruses from the *Orthomyxoviridae* family, cause seasonal epidemics in older adults and can lead to severe complications, especially when vaccine effectiveness is low [7]. *Respiratory syncytial virus (RSV)*, from the *Pneumovirus* genus, is traditionally a pediatric pathogen but has emerged as a significant cause of lower respiratory infections in older adults [8]. *Human metapneumovirus (hMPV)*, also from the *Metapneumovirus* genus, shares clinical similarities with RSV [9]. *Rhinoviruses*, a member of the *Picornaviridae* family, are common upper respiratory pathogens that circulate throughout the year [10].

Despite the clinical significance of these viruses, few long-term studies have analyzed their infection patterns, seasonal variation, and sex-specific differences in older adults. The lack of data hampers the development of evidence-based prevention and vaccination strategies tailored for this population. The coronavirus disease (COVID-19) pandemic has significantly altered the epidemiology and surveillance systems of respiratory viruses. Non-pharmaceutical interventions (NPIs), such as social distancing, masking, and travel restrictions, have disrupted the transmission of seasonal viruses, leading to their temporary disappearance and delayed circulation [11]. Although post-pandemic re-emergence and altered seasonal patterns have been observed, long-term quantitative studies on older adults remain limited [12].

To address this gap, we conducted a retrospective analysis of polymerase chain reaction (PCR)-based respiratory virus surveillance data from a 1000-bed tertiary care hospital over an 18-year period (2007–2024). In this study, we aimed to evaluate infection trends, seasonal variation, and sex-specific differences in respiratory virus positivity among older adults (≥65 years), with a particular focus on changes before and after the COVID-19 pandemic. By providing longitudinal, age-specific data, we aim to inform future infection control strategies and vaccine prioritization in this vulnerable population.

## 2. Materials and Methods

### 2.1. Study Design, Setting, and Study Period

This retrospective cross-sectional study was conducted from 1 January 2007 to 31 December 2024 at a 921bed tertiary care hospital (Dankook University Hospital) in Cheonan, Chungcheongnam-do, Republic of Korea. Patients with acute respiratory symptoms, such as cough, fever, sputum production, or dyspnea, were tested for respiratory viruses at outpatient clinics or inpatient wards.

### 2.2. Study Population

This study included adults aged ≥ 65 years who underwent PCR testing for respiratory viruses due to acute respiratory symptoms between 1 January 2007 and 31 December 2024. The inclusion criteria were as follows: (1) age ≥ 65 years; (2) clinical presentation with cough, fever, or dyspnea; and (3) available PCR test result. The exclusion criteria were as follows: (1) asymptomatic patients tested for screening only, (2) incomplete test data, and (3) repeat tests during the same illness episode.

### 2.3. Sample Collection and Virus Detection

Nasopharyngeal swab specimens were collected from all patients presenting with acute respiratory symptoms, such as cough, fever, sputum production, or dyspnea, either at outpatient clinics or inpatient wards. The specimens were transported to the laboratory under refrigerated conditions (2–8 °C) and tested within 24 h of collection. Respiratory viruses were detected using a commercially available real-time PCR assay (AdvanSure RV; LG Chem, Seoul, Republic of Korea), following the manufacturer’s instructions. The assay targets 15 respiratory viruses, including RNA viruses (genus *Influenza A/B* family *Orthomyxovirus*, genus *RSV A/B* family *Pneumovirus*, genus *hMPV* family *Metapneumovirus*, genus *parainfluenza virus types 1/2/3*; *rhinovirus* family *Enterovirus* and *human coronaviruses 229E, OC*43*,* and *NL*63) and DNA viruses (genus adenovirus family *Mastadenovirus* and genus *bocavirus* family *Bocaparvovirus*). The kit is designed for RNA-based extraction, while also facilitating the simultaneous detection of DNA viruses, such as *human bocavirus (HBoV)* and *adenovirus*. *HBoV* and *NL63* were included in the panel starting in 2015, and enterovirus was added in 2018. In cases where the results were inconclusive or invalid, repeat testing was conducted on the same specimen.

### 2.4. Variables and Classification

All detected viruses were recorded individually. When multiple viruses were detected in a single specimen, each virus was counted as an independent positive case. Positivity rates were calculated on a per-virus basis and may differ from total patient counts. Data were extracted from the hospital’s Laboratory Information System, which maintains all respiratory virus PCR results and patient demographics. Analyses were restricted to older adults (≥65 years), classified according to the ICH E7(R1) guidelines of the International Council for Harmonisation of Technical Requirements for Pharmaceuticals for Human Use (ICH). Variables included annual positivity counts and rates, seasonal distribution (spring: March–May; summer: June–August; autumn: September–November; winter: December–February), and sex-specific infection rates. The study period was divided into three phases: pre-pandemic (2007–2019), pandemic (2020–2022), and post-pandemic (2023–2024).

### 2.5. Statistical Analyses

**Figure 1:**
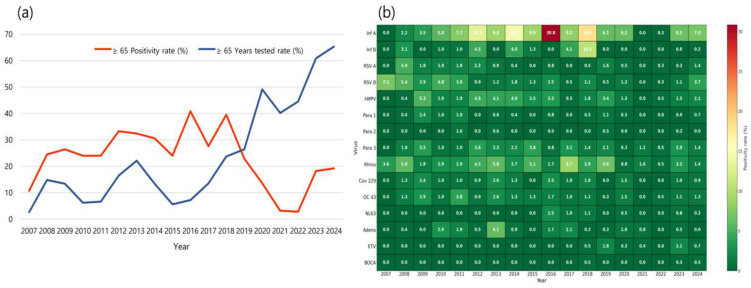
Long-term testing participation, positivity trends, and virus-specific circulation patterns among older adults (≥65 years) in Korea, 2007–2024. (**a**) Annual trends in testing participation and positivity rates among individuals aged ≥ 65 years. The blue line denotes the proportion of older adults among the total tested population, whereas the red line represents the positivity rate within this age group. (**b**) Heatmap of annual positivity rates (%) for 15 respiratory viruses among older adults (≥65 years). Each cell corresponds to the virus-specific positivity rate for a given year. This panel highlights heterogeneous circulation patterns across viruses and years, providing complementary insights to the overall testing and positivity trends shown in panel (**a**). Virus abbreviations: *Inf A/B, Influenza A/B virus; RSV A/B, Respiratory syncytial virus A/B; hMPV, Human metapneumovirus; Para 1/2/3, Parainfluenza virus types 1/2/3; Rhino, Rhinovirus; CoV 229/OC43/NL63, Human coronavirus 229E/OC43/NL63; Adeno, Adenovirus; ETV, Enterovirus; BOCA, Human bocavirus*.

Data were organized using Microsoft Excel, version 2021 (Microsoft Corp., WA, USA), and statistical analyses were performed using R software (version 4.5.1; R Core Team, University of Auckland, Auckland, New Zealand). The differences in positivity rates by year, season, and sex were assessed using independent two-sample *t*-tests and two-tailed Pearson’s chi-square tests. The positivity rates for each of the 15 respiratory viruses were calculated separately. In cases of co-detection, each virus was counted independently, and multiple viruses detected in a single specimen were each included in the respective virus-specific positivity rates. The positivity rate was calculated as the number of patients with laboratory-confirmed virus detection divided by the total number of tests performed in each subgroup (year, season, or sex), expressed as a percentage. In cases of co-detection, each virus was counted independently per detection event. Therefore, the sum of virus-specific positivity rates may exceed the total positivity rate across all viruses. A *p*-value of <0.05 was considered statistically significant. The Figure 1b heatmap was generated to visualize the annual positivity rates of 15 respiratory viruses, with darker red shades indicating higher positivity. Heatmaps were generated to illustrate the seasonal distribution of 15 respiratory viruses. Each cell represents the total number of positive cases for a given virus in each season, with color intensity corresponding to case counts. Darker shades indicate higher incidence, allowing intuitive comparison of viral activity levels across seasons.

## 3. Results

### 3.1. Participant Characteristics

A total of 4692 respiratory virus PCR test records from individuals aged ≥ 65 years were included in the final analysis. This study population represents symptomatic older adults who presented to a tertiary care hospital for inpatient or outpatient evaluation during the study period.

Of the 4692 participants, 3036 (64.7%) were male and 1656 (35.3%) were female. The median age was 77 years, with an interquartile range (IQR) of 71–82 years. Specimens were collected from multiple clinical departments, including internal medicine, emergency medicine, and pulmonology.

Based on the testing period, 2627 (56.0%) cases occurred in the pre-pandemic period (2007–2019), 1019 (21.7%) during the pandemic (2020–2022), and 1046 (22.3%) in the post-pandemic period (2023–2024).

The highest number of samples was collected in winter (*n* = 1436, 30.6%), followed by spring (*n* = 1221, 26.0%), autumn (*n* = 1016, 21.7%), and summer (*n* = 1019, 21.7%).

### 3.2. Trends in Testing Participation and Positivity Rates Among Older Adults, 2007–2024

From 2007 to 2024, the proportion of respiratory virus PCR tests conducted on older adults at the study institution showed a significant increase (*p* < 0.0001) from 2007 to 2024. During the pre-pandemic period (2007–2019), only 13.2% of all individuals tested were older adults. However, following the onset of the COVID-19 pandemic (2020–2024), this proportion surged to an average of 52.0%, with a peak of 65.3% in 2024 alone (Figure 1a). Although the proportion of older adults tested increased after 2020 (mean: 52.0%), the absolute number of tests in older adults was lower than that in the pre-pandemic period (2007–2019: average: 1488 tests vs. 2020–2024: average: 786 tests), indicating that the observed proportion reflects a relative shift rather than an absolute increase in testing volume.

Despite the sharp increase in testing participation, the annual positivity rate among older adults declined significantly after the pandemic began (*p* < 0.0001). The average positivity rate during the pre-pandemic period was 27.7%, but dropped to 11.4% in the pandemic and post-pandemic years. The lowest positivity rates were observed in 2021 (3.2%) and 2022 (2.8%) (Table S1).

To further delineate virus-specific dynamics, we additionally analyzed the annual positivity rates for each of the 15 respiratory viruses in individuals aged ≥ 65 years (Figure 1b).

### 3.3. Detection of Respiratory Viruses in Older Adults Before, During, and After the COVID-19 Pandemic

When considering the entire study period (2007–2024), the most frequently identified pathogen in older adults was *influenza A* virus (*n* = 374, 32.2%), followed by *rhinovirus* (*n* = 159, 13.7%) and *hMPV* (*n* = 100, 8.6%). *Influenza A* was significantly more prevalent than the other viruses (*p* < 0.0001). Distinct shifts in virus detection patterns were observed before, during, and after the COVID-19 pandemic.

- Pre-pandemic period (2007–2019): Stable circulation of seasonal viruses

**Figure 2:**
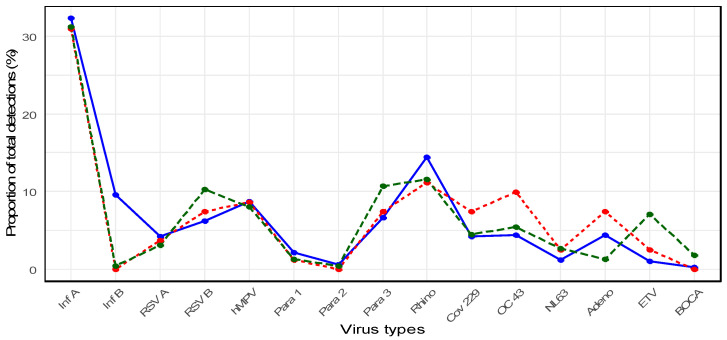
Annual distribution of respiratory virus detections in older adults (≥65 years) during the pre-pandemic (2007–2019), pandemic (2020–2022), and post-pandemic (2023–2024) periods. The line graph illustrates the proportion of total detections (%) for 15 respiratory viruses among older adults (≥65 years). The blue line indicates the pre-pandemic period (2007–2019), the red line represents the pandemic period (2020–2022), and the green line represents the post-pandemic period (2023–2024). Virus abbreviations: *Inf A/B: Influenza A/B virus, RSV A/B: Respiratory syncytial virus A/B, hMPV: Human metapneumovirus, Para 1/2/3: Parainfluenza virus types 1/2/3, Rhino: Rhinovirus, CoV 229/OC43/NL63: Human coronavirus 229E/OC43/NL63, Adeno: Adenovirus, ETV: Enterovirus, BOCA: Human bocavirus.* Note: *Enterovirus* was included in the diagnostic panel starting in 2018, whereas *human bocavirus* and *coronavirus NL63* were added in 2015. Therefore, earlier years may show no detections because of the absence of corresponding assays in the testing platform at that time.

During the pre-pandemic years, older adults exhibited regular seasonal circulation of respiratory viruses (Figure 2). *Influenza A* was the predominant pathogen, with a proportion of 32.3% among all viral detections, followed by *rhinovirus* (14.4%), *influenza B* (9.6%), *hMPV* (8.7%), *parainfluenza virus type* 3 (6.6%), and *RSV B* (6.2%). Other viruses such as *adenovirus* (4.4%), *OC*43 (4.4%), 229*E* (4.2%), and *RSV A* (4.2%) circulated at lower proportions, while *parainfluenza* 1 (2.1%), *NL*63 (1.2%), *enterovirus* (1.0%), *parainfluenza* 2 (0.6%), and *human bocavirus* (0.2%) were only sporadically detected. Notably, in 2018, *influenza A* showed a marked surge, accounting for 21.3% (80/374) of all *influenza A* detections during the entire study period (Table S2).

- Pandemic period (2020–2022): Dramatic suppression of the circulation of the virus

Compared with the pre-pandemic period, the total number of virus detections declined substantially. *Influenza A* remained the most frequently detected virus during the pandemic (30.9%), followed by *rhinovirus* (11.1%), *OC43* (9.9%), and *hMPV* (8.6%), while other viruses such as *RSV B*, *adenovirus*, and *parainfluenza virus type 3* each accounted for only 7.4% of detections (Figure 2). In 2021, no cases of *influenza A/B* or *RSV A/B* were identified, and the detection of other viruses was nearly absent, reflecting the widespread suppression of viral transmission during the pandemic (Table S2).

- Post-pandemic period (2023–2024): Re-emergence of respiratory viruses

Several respiratory viruses began to re-emerge in older adults following the pandemic. In 2024, *RSV B* cases increased to 3.7% (16/427), whereas *influenza A* cases rose to 7.0% (30/427). In 2023, *rhinovirus* cases accounted for 3.2% (20/619), suggesting a gradual return to pre-pandemic circulation patterns (Table S2).

Overall, *influenza A* remained the most prevalent virus during the post-pandemic period (31.2%), followed by *rhinovirus* (11.6%), *parainfluenza virus type 3* (10.7%), *RSV B* (10.3%), and *hMPV* (8.0%) (Figure 2).

### 3.4. Seasonal Patterns of Respiratory Virus Infections in Older Adults

An analysis of the seasonal respiratory virus positivity rates among older adults from 2007 to 2024 revealed the highest positivity during winter, reaching 34.4%, which was significantly higher than that in other seasons (*p* < 0.0001). This was followed by spring (26.1%), summer (12.1%), and autumn (11.6%) (Table S3).

- Pre-pandemic period (2007–2019)

**Figure 3:**
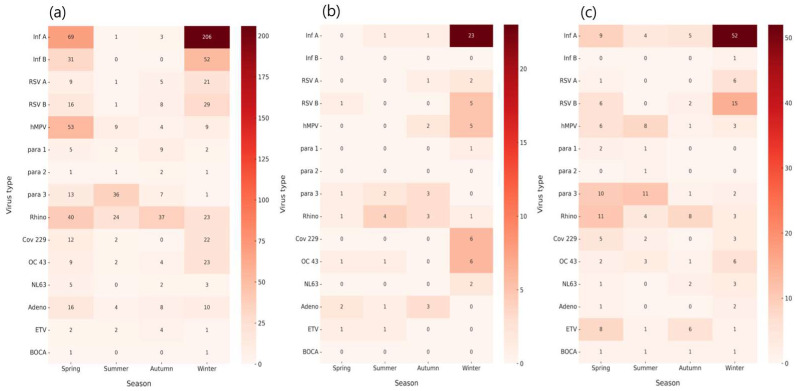
Seasonal distribution of respiratory virus detections among older adults (≥65 years) in Korea during the pre-pandemic, pandemic, and post-pandemic periods (2007–2024). (**a**) Seasonal distribution of respiratory virus–positive cases during the pre-pandemic period (2007–2019). (**b**) Seasonal distribution of respiratory virus–positive cases during the COVID-19 pandemic (2020–2022). (**c**) Seasonal distribution of respiratory virus–positive cases during the post-pandemic period (2023–2024). This panelized visualization demonstrates distinct seasonal circulation patterns across the pre-, peri-, and post-pandemic periods, highlighting the influence of public health interventions and the subsequent re-emergence of respiratory viruses. Virus abbreviations: *Inf A/B, Influenza A/B virus; RSV A/B, Respiratory syncytial virus A/B; hMPV, Human metapneumovirus; Para 1/2/3, Parainfluenza virus types 1/2/3; Rhino, Rhinovirus; CoV 229/OC43/NL63, Human coronavirus 229E/OC43/NL63; Adeno, Adenovirus; ETV, Enterovirus; BOCA, Human bocavirus*.

During the pre-pandemic period, clear seasonality was observed. *Influenza A* and *B* occurred predominantly in winter (*influenza A*: *n* = 206, *influenza B*: *n* = 52), and *RSV A* (*n* = 21) and *RSV B* (*n* = 29) also peaked in winter. In contrast, *para 3* showed a distinct summer peak (*n* = 36), while *hMPV* was most frequently detected in spring (*n* = 53). *Rhinovirus* circulated year-round with relatively even distribution across all seasons (spring: 40, summer: 24, autumn: 37, winter: 23). *Coronaviruses (OC43, 229E)* were mainly detected in winter, and adenovirus was found across all seasons. Low-prevalence viruses (*NL63, enterovirus, bocavirus*) showed no consistent seasonal pattern (Figure 3a).

- Pandemic period (2020–2022)

During the pandemic period, respiratory virus circulation was markedly suppressed across all seasons. *Influenza A* was detected almost exclusively in winter (*n* = 23), with only sporadic detections in summer (*n* = 1) and autumn (*n* = 1). No cases of *influenza B* were observed. *RSV A* (*n* = 3) and *RSV B* (*n* = 6) showed minimal circulation, confined primarily to winter. *hMPV* exhibited limited detections in autumn (*n* = 2) and winter (*n* = 5). *Para 3* showed small numbers across summer (*n* = 2) and autumn (*n* = 3), while *rhinovirus* persisted throughout all seasons at very low levels (spring: *n* = 1, summer: *n* = 4, autumn: *n* = 3, winter: *n* = 1). *OC43* was mainly detected in winter (*n* = 6), whereas *229E, NL63*, and *adenovirus* were only sporadically identified. *Enterovirus (ETV)* was detected at very low levels (spring and summer: *n* = 1 each). No cases of bocavirus or *parainfluenza virus type 2* were recorded. Overall, these findings highlight the dramatic suppression of most respiratory viruses during the pandemic, with *influenza*, *RSV*, and *hMPV* activity reduced to sporadic detections and rhinovirus representing the only virus with year-round, albeit minimal presence (Figure 3b).

- Post-pandemic period (2023–2024)

In the post-pandemic years, the circulation of respiratory viruses in older adults showed clear signs of re-emergence. *Influenza A* re-established strong winter seasonality, with the highest detections in winter (*n* = 52), while only sporadic cases occurred in spring (*n* = 9), summer (*n* = 4), and autumn (*n* = 5). *Influenza B* was nearly absent, with only one case reported in winter.

*RSV A* (*n* = 6) and *RSV B* (*n* = 15) displayed a distinct winter peak, with additional minor detections in spring and autumn. *hMPV* exhibited a broader distribution, with the highest detections in summer (*n* = 8), followed by spring (*n* = 6) and winter (*n* = 3). *Parainfluenza virus type 3* circulated primarily in spring (*n* = 10) and summer (*n* = 11), with fewer cases in autumn (*n* = 1) and winter (*n* = 2), confirming its characteristic warm-season activity. *Rhinovirus* persisted year-round, with cases across all seasons (spring: *n* = 11, summer: *n* = 4, autumn: *n* = 8, winter: *n* = 3).

Seasonal detection of *coronaviruses* was also evident: *OC43* peaked in winter (*n* = 6), while *229E* appeared in both spring (*n* = 5) and winter (*n* = 3). *NL63* was sporadically detected across multiple seasons, with small clusters in autumn (*n* = 2) and winter (*n* = 3). Adenovirus and enterovirus were identified at low frequencies, with enterovirus showing a broader distribution (spring: *n* = 8, autumn: *n* = 6). Human bocavirus remained rare, with one case detected in each season (Figure 3c).

Overall, these findings indicate a gradual return toward pre-pandemic seasonal patterns, with *influenza A*, *RSV*, and *OC43* re-establishing their winter predominance, *parainfluenza 3* showing summer peaks, and *rhinovirus* maintaining year-round circulation.

### 3.5. Sex-Specific Differences in Respiratory Virus Infections Among Older Adults

From 2007 to 2024, a total of 4692 respiratory virus PCR tests were performed in older adults, with 3036 tests in men (positive cases: *n* = 635, 20.9%) and 1656 tests in women (positive cases: *n* = 420, 25.3%). The overall positivity rate was significantly higher in women than in men (*p* = 0.0005).

As shown in Table S4, *influenza A* was significantly more common in women (9.7%) than in men (7.0%) (*p* = 0.0013). Although the number of *influenza B* cases was equal between sexes, the positivity rate was higher in women due to a lower number of total tests, with a statistically significant difference observed (*p* = 0.0063). In contrast, no significant sex-specific differences were observed in the positivity rates of the other 13 viruses, including *RSV A/B, hMPV, parainfluenza virus types 1/2/3, rhinovirus, human coronaviruses 229E, OC43*, and *NL63, adenovirus, enterovirus*, and *human bocavirus* (*p* > 0.05).

When stratified by study period, sex-specific differences were most apparent for *influenza A* and *B*.

- Pre-pandemic period (2007–2019)

Women generally exhibited higher positivity rates for both *influenza A* and *B*. Nonetheless, certain exceptions were observed in which men showed higher rates, including *influenza A* in 2008, 2010, and 2015, and *influenza B* in 2010 and 2011. Notably, years with no detected positive cases resulted in a recorded positivity rate of 0%, such as *influenza A* in 2007 and *influenza B* in 2007, 2009, 2013, 2016, and 2019. Statistical testing revealed that these sex differences were not significant in most years, with the exception of *influenza A* in 2018 (*p* = 0.034) and *influenza B* in 2014 (*p* = 0.021), where women demonstrated significantly higher positivity rates compared to men.

- Pandemic period (2020–2022)

During the pandemic period (2020–2022), *influenza A* circulation markedly declined, with only sporadic detection in 2020 (5.6% in men vs. 7.2% in women), and no cases recorded in 2021. The observed sex difference in 2020 was not statistically significant (*p* = 0.722). *Influenza B* was completely absent during this interval, precluding any sex- specific comparisons.

- Post-pandemic period (2023–2024)

A female predominance in influenza A re-emerged, with higher positivity in women than in men (2023: 6.7% vs. 6.2%, *p* = 0.948; 2024: 9.1% vs. 5.9%, *p* = 0.311). *Influenza B* activity remained minimal, with only sporadic cases detected (2024: 0.3% in men vs. 0% in women), and this difference was not significant (*p* = 1.000).

**Figure 4:**
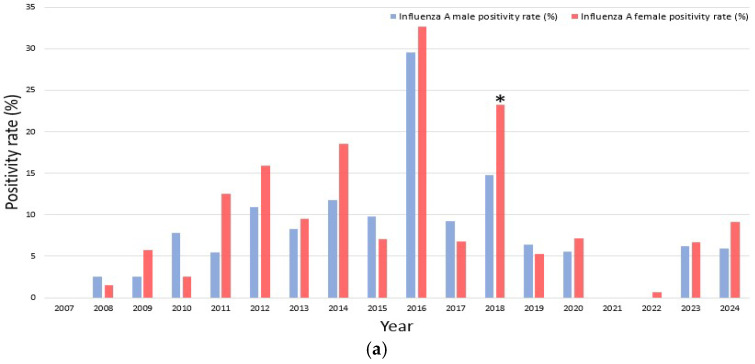

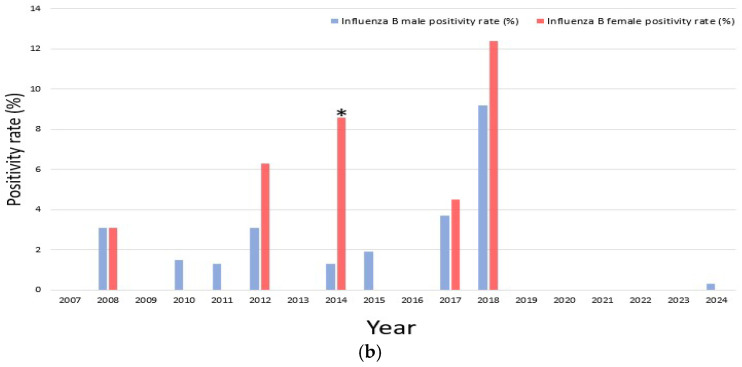
(**a**). Annual sex-specific positivity rates of *influenza A* (2007–2024). The bar chart depicts yearly positivity rates for *influenza A* in male (blue) and female (red), with asterisks (* *p* < 0.05). (**b**) Annual sex-specific positivity rates of *influenza B* (2007–2024). The bar chart depicts yearly positivity rates for *influenza B* in male (blue) and female (red), with asterisks (* *p* < 0.05).

Overall, women tended to show higher positivity rates for both *influenza A* and *B*; however, inter-annual variability was evident. These findings are illustrated in Figure 4a (*influenza A*) and Figure 4b (*influenza B*).

## 4. Discussion

Based on 18 years of clinical surveillance data from a single tertiary care center, this study provides a rare longitudinal perspective on respiratory virus infections in older adults, with a particular focus on the global turning point in infectious disease dynamics—the epidemiological shifts surrounding the COVID-19 pandemic.

Although older adults accounted for a small fraction of those tested for respiratory viruses before the pandemic, their participation increased substantially during and after the pandemic. This shift reflects a change in the redistribution of clinical priorities and healthcare resources toward high-risk populations [13]. Increased clinical vigilance and broad screening strategies led to a greater inclusion of older adults in surveillance programs following the emergence of COVID-19 [14]. This transformation signifies increased healthcare use and a strategic repositioning of older adults as a central focus in public health surveillance.

Interestingly, despite the increased testing volume, positivity rates declined significantly during the pandemic. This decline likely reflects a dilution effect from relaxed testing criteria, influenza vaccination, and effectiveness of NPIs, such as masking and social distancing [15,16,17,18]. Consistent patterns have been observed in multiple studies conducted in the United States and Europe, which similarly reported a near-complete disappearance of influenza and RSV activity during the pandemic period due to the widespread implementation of NPIs [19,20].

Despite the relatively lower number of tests conducted between 2020 and 2024 compared to previous years, a consistent decline was observed across multiple respiratory viruses. The suppression patterns evident in seasonal heatmaps and the re-emergence of certain viruses after 2023 further support the robustness of our findings. These results suggest that the observed trends are not solely attributable to a reduction in testing volume but likely reflect genuine epidemiological shifts driven by public health interventions and changes in population-level immunity dynamics.

Future studies should consider additional factors, such as changes in test-seeking behavior and healthcare accessibility during the pandemic period, which may have influenced virus detection trends.

Given that even mild infections in older adults can progress to serious complications, the combination of widespread testing and effective NPIs is crucial for ensuring clinical preparedness and minimizing disease burden in this age group [21].

Seasonal variation in virus circulation was evident in this study, indicating that respiratory virus ecological characteristics are preserved even in older populations. *Influenza A*, *RSV A/B*, and seasonal *coronaviruses (229E, OC43)* were predominantly detected in winter, emphasizing the importance of aligning vaccine campaigns and preparedness systems with seasonal epidemiology [22,23,24]. In contrast, *para 3* peaked in summer, and *hMPV* showed relatively even distribution across spring and summer—suggesting that some viruses may circulate at seasonal boundaries [25,26]. *Rhinovirus* has been consistently detected throughout all seasons, reinforcing its role as a year-round pathogen even in older adults [10]. Post-pandemic shifts in the timing and distribution of virus circulation may be attributed to weakened population-level immunity, residual effects of NPIs, and “immune debt” resulting from disrupted immune memory [27]. The continuous detection of non-influenza viruses with varying seasonality highlights the need to expand surveillance frameworks to include a broader spectrum of pathogens.

In addition to these seasonal differences, many respiratory viruses are known to follow biennial circulation patterns. Likewise, our long-term data revealed alternating strong and weak epidemic years, particularly for *RSV* and *PIV3*, resembling the well-documented “2-year rhythm” phenomenon. This observation may reflect population-level immunity dynamics, such as the accumulation and waning of susceptible individuals, and suggests the need for future multicenter studies to validate these biennial patterns in older adults.

The observed sex- specific differences, namely the higher positivity rates of *influenza A* and *B* in women, are likely associated with immunological and physiological factors rather than viral tropism. Previous studies have reported that women exhibit enhanced innate immune responses, including stronger interferon signaling, and that sex hormones such as estrogen and progesterone play important regulatory roles in antiviral immunity [28,29]. When the entire study period was combined, women showed significantly higher positivity rates for *influenza A* and *B*; however, year-by-year analyses revealed statistical significance only in certain years, while other years did not reach significance. This inter-annual variability may reflect differences in virus circulation intensity, sample size limitations, and population-level immunity, although the repeated trend of female predominance observed across multiple periods suggests that these differences are unlikely to be incidental. Taken together, these findings indicate that biological mechanisms, coupled with temporal variability, may underlie the higher susceptibility of older women to influenza infections, although further research is warranted.

In line with the ICH E7(R1) guideline, this study defined older adults (≥65 years) as a distinct analytical cohort, recognizing the intrinsic age-related susceptibility to infections [30]. Although several studies have focused on pediatric populations, our study aimed to address an important gap by establishing an independent surveillance framework for older adults [31]. Given the increasing recognition of influenza as a major contributor to the respiratory disease burden in this demographic, the seasonal and sex-stratified findings presented here emphasize the critical need for targeted surveillance in older adult populations [32].

Despite the strengths of this study’s long-term time-series analysis, certain limitations must be acknowledged. First, the inclusion of *HBoV* and coronavirus *NL63* in 2015 and enterovirus in 2018 may have influenced the pre-pandemic virus-specific detection rates. For example, *HBoV* and *NL63* were not detected in earlier years simply because of the lack of circulation and not because of the absence of diagnostic capability. Therefore, comparisons of detection rates before and after inclusion are not possible, and trends may reflect testing availability rather than true epidemiological shifts. Second, the presence of clinical variables, such as disease severity, hospitalization status, comorbidities, and vaccination history, may have introduced spectrum bias. Third, environmental, behavioral, and socioeconomic factors (temperature, humidity, population density, and healthcare access) were not assessed, limiting the ability to control for potential confounders. In addition, although the testing volume was considered, unmeasured pandemic-related factors such as changes in test-seeking behavior or healthcare accessibility may have also influenced the observed patterns.

Fourth, co-infection data were not included, which may have underestimated the disease burden due to viral interactions. In addition, genotypic data were unavailable, raising the possibility of generalization errors, particularly in interpreting *HBoV*, where *type 1* is typically dominant. Furthermore, although the study defined older adults as individuals aged ≥ 65 years in accordance with the ICH E7(R1) guideline, this age threshold may differ from those used in other studies (e.g., ≥60 or ≥70 years), potentially limiting cross-study comparability.

Despite these limitations, the overall implications for the design of surveillance systems and long-term monitoring remain robust. Future research should aim to establish multicenter, nationwide cohorts to validate the applicability of these findings across diverse regions of Korea and other temperate Asian settings. Linking epidemiological trends to clinical outcomes, such as hospitalization, vaccination history, and mortality, will facilitate the development of evidence-based public health policies tailored to older adults. In addition, co-infections involving multiple respiratory viruses were observed in a subset of patients in this dataset. A detailed analysis of co-infection patterns and their clinical implications is warranted in future studies.

## 5. Conclusions

In this study, we quantitatively analyzed the long-term trends in respiratory virus infections among older adults (≥65 years) using standardized surveillance data from 18 years. We provide a deeper understanding of age-specific infectious disease epidemiology by comprehensively evaluating seasonal patterns, sex-specific susceptibility, and structural changes in viral circulation before and after the COVID-19 pandemic.

Our findings highlight the circulation dynamics and immunological susceptibility of major respiratory viruses—*influenza A*, *RSV*, *hMPV*, and *para* 3—within the older adult population. These results offer valuable insights into virus-specific pathophysiology, host immune responses, and the impact of factors such as waning immunity and immune debt in this vulnerable group. By extending the epidemiological focus beyond pediatric populations, this study lays the groundwork for precision epidemiology and pathogenetic research targeting older adults.

Moreover, the quantification of seasonal variability and shifts in viral circulation before and after the pandemic holds promise for advancing future research on surveillance system design, host–pathogen interactions, and the relationship between viral genotypes and host immunity. This study provides empirical evidence to inform the development of age-targeted infectious disease response strategies based on a thorough assessment of structural changes in viral epidemiology and differential susceptibility by age, sex, and season.

In conclusion, respiratory virus epidemiology in older adults underwent significant changes following the COVID-19 pandemic. This study offers quantitative evidence of these shifts and establishes a foundation for long-term surveillance, targeted vaccination strategies, and proactive preparedness planning for this high-risk population. Given their distinct epidemiological characteristics, older adults warrant classification as a standalone surveillance group. To ensure timely detection and effective response, surveillance systems should remain flexible, adapt to real-time changes in infection patterns, and optimize testing strategies, target populations, and resource allocation based on annual variations in epidemic onset.

## Data Availability

The data that support the findings of this study are derived from patient records at Dankook University Hospital and are subject to ethical and legal restrictions. Due to privacy and confidentiality concerns, the raw datasets cannot be made publicly available. However, anonymized summary data are available from the corresponding author upon reasonable request, subject to approval by the Institutional Review Board.

## Supplementary Materials

The following supporting information can be downloaded at: https://www.mdpi.com/article/10.3390/microorganisms13102301/s1, Table S1: Annual number of tested individuals and positivity rates for respiratory viruses among older adults (≥65 years) from 2007 to 2024; Table S2: Annual counts of respiratory viruses detected in patients aged ≥65 years (2007–2024), Table S3: Seasonal distribution of respiratory virus detections among older adults (≥65 years) from 2007 to 2024, Table S4: Sex-specific positivity rates and statistical significance for respiratory viruses among older people (≥65 years), 2007–2024.

## Abbreviations

The following abbreviations are used in this manuscript:

RSV	Respiratory syncytial virus
hMPV	Human metapneumovirus
COVID-19	Coronavirus disease 2019
NPIs	Non-pharmaceutical interventions
PCR	Polymerase chain reaction
HBoV	Human bocavirus
ICH	International Council for Harmonisation of Technical Requirements for Pharmaceuticals for Human Use
IRB	Institutional Review Board
IQR	Interquartile range
Inf A/B	Influenza A/B virus
Para 1/2/3	Parainfluenza virus types 1/2/3
Rhino	Rhinovirus
Adeno	Adenovirus
ETV	Enterovirus
BOCA	Human bocavirus
