# Supplementary material for: Epidemiological Shifts in Respiratory Virus Infections Among Older Adults (≥65 Years) Before and After the COVID-19 Pandemic: An 18-Year Retrospective Study in the Republic of Korea
Source: Microorganisms. 2025 Oct 3;13(10):2301. doi: 10.3390/microorganisms13102301 (PMC12566155; doi:10.3390/microorganisms13102301)
Supplement: Supplementary file 1 [file microorganisms-13-02301-s001.zip › microorganisms-3869076-supplementary/Infections_in_Older_Adults_≥65_Years-Table_S4.pdf]

Table S4 Sex-specific positivity rates and statistical significance for respiratory viruses among older people (≥ 65 years), 2007–2024

Virus	Positive (n)		Positivity rate (%)		*p*-value
	Men	Women	Men	Women	
Inf A	213	161	7.0	9.7	0.0013
Inf B	42	42	1.3	2.5	0.0063
RSV A	26	20	0.8	1.2	0.3114
RSV B	53	30	1.7	1.8	0.9619
hMPV	59	41	1.9	2.4	0.2708
Para 1	16	6	0.5	0.3	0.5717
Para 2	2	4	0.06	0.2	0.2373
Para3	48	39	1.5	2.3	0.0776
Rhino	103	56	3.3	3.3	1.0000
CoV					
229	30	22	0.9	1.3	0.3584
OC43	44	14	1.4	0.8	0.0988
NL63	15	3	0.4	0.1	0.1586
Adeno	30	17	0.9	1.0	1.0000
ETV	17	10	0.5	0.6	1.0000
Boca	4	2	0.1	0.1	1.0000

This table summarizes the number of positive cases and positivity rates for each respiratory virus among individuals aged ≥ 65 years. The positivity rate was calculated as the proportion of positive cases among the total number of tests conducted for each sex. Statistical significance was assessed using the chi-square test. Influenza A and B exhibited significantly higher positivity rates in women than in men.

Virus abbreviations: Inf A/B: Influenza A/B virus, RSV A/B: Respiratory syncytial virus A/B, hMPV: Human metapneumovirus, Para 1/2/3: Parainfluenza virus types 1/2/3, Rhino: Rhinovirus, CoV 229/OC43/NL63: Human coronavirus 229E/OC43/NL63, Adeno: Adenovirus, ETV: Enterovirus, Boca: Human bocavirus.
