# Supplementary material for: Epidemiological Shifts in Respiratory Virus Infections Among Older Adults (≥65 Years) Before and After the COVID-19 Pandemic: An 18-Year Retrospective Study in the Republic of Korea
Source: Microorganisms. 2025 Oct 3;13(10):2301. doi: 10.3390/microorganisms13102301 (PMC12566155; doi:10.3390/microorganisms13102301)
Supplement: Supplementary file 1 [file microorganisms-13-02301-s001.zip › microorganisms-3869076-supplementary/Infections_in_Older_Adults_≥65_Years-_Table_S1.pdf]

Table S1 Annual number of tested individuals and positivity rates for respiratory viruses among older adults (≥ 65 years) from 2007 to 2024.

Year	Total tested (n)	≥ 65 years		
		Tested (n)	Positive (n)	Positivity rate (%)
2007	1,057	28	3	10.7
2008	1,504	224	55	24.5
2009	1,265	170	45	26.4
2010	1,657	104	25	24.0
2011	1,568	104	25	24.0
2012	1,345	222	74	33.3
2013	1,545	342	111	32.4
2014	1,674	225	69	30.6
2015	1,388	79	19	24.0
2016	1,645	120	49	40.8
2017	1,436	195	54	27.6
2018	1,834	435	172	39.5
2019	1,432	379	87	22.9
2020	792	389	53	13.6
2021	613	247	8	3.2
2022	860	383	11	2.8
2023	1,016	619	113	18.2
2024	653	427	82	19.2
