# Supplementary material for: Epidemiological Shifts in Respiratory Virus Infections Among Older Adults (≥65 Years) Before and After the COVID-19 Pandemic: An 18-Year Retrospective Study in the Republic of Korea
Source: Microorganisms. 2025 Oct 3;13(10):2301. doi: 10.3390/microorganisms13102301 (PMC12566155; doi:10.3390/microorganisms13102301)
Supplement: Supplementary file 1 [file microorganisms-13-02301-s001.zip › microorganisms-3869076-supplementary/Infections_in_Older_Adults_≥65_Years-_Table_S2.pdf]

Table S2 Annual counts of respiratory viruses detected in patients aged ≥ 65 years (2007–2024)

Year	Inf A	Inf B	RSV A	RSV B	hMPV	Para 1	Para 2	Para 3	Rhino	Cov 229	OC 43	NL 63	Adeno	ETV	BOCA
2007	0	0	0	2	0	0	0	0	1	0	0	0	0	0	0
2008	5	7	11	12	1	1	0	4	13	3	3	0	1	0	0
2009	6	0	3	5	9	4	0	6	3	4	5	0	0	0	0
2010	6	1	2	5	2	1	0	1	3	1	1	0	3	0	0
2011	8	1	2	4	2	2	1	1	3	1	4	0	2	0	0
2012	29	10	5	2	10	0	0	8	10	2	2	0	1	0	0
2013	30	0	3	4	14	2	2	8	20	9	9	0	21	0	0
2014	32	9	1	4	9	1	0	5	6	3	3	0	2	0	0
2015	7	1	0	1	2	0	0	3	4	0	1	0	0	0	0
2016	37	0	1	3	4	1	0	1	2	3	2	3	2	0	0
2017	16	8	0	1	1	0	0	6	17	2	2	2	4	0	0
2018	80	46	2	9	8	2	0	6	17	8	5	5	1	2	2
2019	23	0	6	2	13	4	2	8	25	0	1	0	1	7	0
2020	24	0	2	5	5	1	0	1	3	6	6	2	4	1	0
2021	0	0	0	0	0	0	0	3	4	0	0	0	2	1	0
2022	1	0	1	1	2	0	0	2	2	0	2	0	0	0	0
2023	40	0	1	7	9	0	1	18	20	6	7	5	3	13	2

2024	30	1	6	16	9	3	0	6	6	4	5	1	0	3	2
Total (n)	374	84	46	83	100	22	6	87	159	52	58	18	47	27	6

This table presents the yearly number of positive detections for each respiratory virus among patients aged ≥ 65 years, identified via multiplex PCR testing from 2007 to 2024. A total of 15 virus types were included: Influenza A virus (Inf A); Influenza B virus (Inf B); respiratory syncytial virus A and B (RSV A/B); human metapneumovirus (hMPV); parainfluenza virus types 1–3 (Para 1/2/3); rhinovirus (Rhino); human coronavirus subtypes 229E, OC43, and NL63 (CoV 229, OC43, NL63); adenovirus (Adeno); enterovirus (ETV); and human bocavirus (BOCA). The bottom row indicates the cumulative number of detections for each virus across the 18-year period.

Note: Enterovirus was included in the diagnostic panel starting in 2018, whereas human bocavirus and coronavirus NL63 were added in 2015. Therefore, earlier years may show no detections due to the absence of corresponding assays in the testing platform at that time.
