# Supplementary material for: Epidemiological Shifts in Respiratory Virus Infections Among Older Adults (≥65 Years) Before and After the COVID-19 Pandemic: An 18-Year Retrospective Study in the Republic of Korea
Source: Microorganisms. 2025 Oct 3;13(10):2301. doi: 10.3390/microorganisms13102301 (PMC12566155; doi:10.3390/microorganisms13102301)
Supplement: Supplementary file 1 [file microorganisms-13-02301-s001.zip › microorganisms-3869076-supplementary/Infections_in_Older_Adults_≥65_Years-_Table_S3.pdf]

Table S3. Seasonal distribution of respiratory virus detections among older adults (≥ 65 years) from 2007 to 2024

Season	Elderly tested (n)	Elderly positive (n)	Positivity rate (%)
Spring	1,221	319	26.1
Summer	1,019	124	12.1
Autumn	1,016	118	11.6
Winter	1,436	494	34.4

The seasonal positivity rate was calculated as the number of older patients who tested positive for respiratory viruses divided by the total number tested within each season, expressed as a percentage.
